# *In Silico Veritas*: The Pitfalls and Challenges of Predicting GPCR-Ligand Interactions

**DOI:** 10.3390/ph4091196

**Published:** 2011-09-01

**Authors:** Luc Roumen, Marijn P.A. Sanders, Bas Vroling, Iwan J.P. de Esch, Jacob de Vlieg, Rob Leurs, Jan P.G. Klomp, Sander B. Nabuurs, Chris de Graaf

**Affiliations:** 1 Department of Medicinal Chemistry, VU University Amsterdam, De Boelelaan 1081, Amsterdam 1081 HV, The Netherlands; E-Mails: l.roumen@vu.nl (L.R.); i.de.esch@vu.nl (I.J.P.E.); r.leurs@vu.nl (R.L.); 2 CMBI, NCMLS, Radboud University Nijmegen Medical Centre, Geert Grooteplein Zuid 26-28, Nijmegen 6525 GA, The Netherlands; E-Mails: m.sanders@cmbi.ru.nl (M.P.A.S.); b.vroling@cmbi.ru.nl (B.V.); j.de.vlieg@cmbi.ru.nl (J.V.); s.nabuurs@cmbi.nl (S.B.N.); 3 Department of Molecular Design and Informatics, MRL, MSD, Oss 2031 BN, The Netherlands; E-Mail: j.klomp@cmbi.ru.nl (J.P.G.K.)

**Keywords:** comparative modeling, ligand binding mode prediction, G protein-coupled receptor (GPCR), GPCR Dock 2010

## Abstract

Recently the first community-wide assessments of the prediction of the structures of complexes between proteins and small molecule ligands have been reported in the so called GPCR Dock 2008 and 2010 assessments. In the current review we discuss the different steps along the protein-ligand modeling workflow by critically analyzing the modeling strategies we used to predict the structures of protein-ligand complexes we submitted to the recent GPCR Dock 2010 challenge. These representative test cases, focusing on the pharmaceutically relevant G Protein-Coupled Receptors, are used to demonstrate the strengths and challenges of the different modeling methods. Our analysis indicates that the proper performance of the sequence alignment, introduction of structural adjustments guided by experimental data, and the usage of experimental data to identify protein-ligand interactions are critical steps in the protein-ligand modeling protocol.

## 1. Introduction

In the last few years, the disciplines involved in *in silico* protein structure prediction have greatly evolved. Not only have more tools and modeling programs become available, but also the amount of varying approaches to produce predictive models has increased. Structure prediction of protein-ligand complexes by comparative or homology modeling can be subdivided into the following major steps: (1) identification of homologue proteins for which a three-dimensional structure is available; (2) alignment of the target sequence with the sequence of the template structure; (3) building the coordinates of the three-dimensional model of the target; (4) modeling the protein-ligand interactions; and (5) assessing ligand binding mode prediction accuracy by investigating ligand structure activity data or biological data [1,2].

Critical assessments of *in silico* methods to predict the structure of proteins (CASP [3]) and protein-protein complexes (CAPRI [4]) have been established in the past years, and many comparative docking studies to predict the binding orientation of small molecule ligands in known protein structures have been reported [5]. Only very recently, however, the first community-wide assessments of the prediction of the structures of complexes between proteins and small molecule ligands have been reported in the so-called GPCR Dock assessments [6,7]. The first was initiated in 2008 to predict conformation of the human adenosine A2A receptor in complex with the small ligand ZM241385 [7,8]. The second was organized in 2010 [6] to predict the conformation of the dopamine D3 receptor in complex with the small ligand eticlopride [9], as well as the chemokine receptor CXCR4 bound to the small ligand 1t [10] or the cyclic peptide CVX15 [6,10]. These assessments did not only give the protein modeling community the chance to objectively (and prospectively) test their methods to predict the structure of complexes between proteins and small (drug-like) ligands, but also offered a unique opportunity to identify the problems and pitfalls in the prediction of protein-ligand interactions. In the current review we will discuss the different steps along the protein-ligand modeling workflow by critically analyzing the modeling strategies we used to generate the structures we submitted to the GPCR Dock 2010 challenge. These representative test cases will be used to demonstrate the strengths and challenges of the different methodologies and their impact on modeling accuracy.

## 2. Experimental Section

### 2.1. GPCR Dock 2010

**Figure 1:**
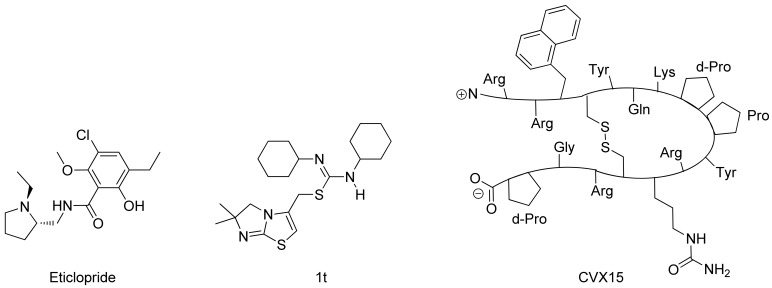
Chemical structures of eticlopride, 1t and CVX15. The first is co-crystallized with the dopamine D3 Receptor and the latter two with the chemokine Receptor CXCR4.

In spring 2010, the group of Stevens *et al.* challenged the scientific community to participate in the structure prediction assessment GPCR Dock 2010. The subject of the challenge consisted of three different crystal structures for which multiple models could be deposited. The first case encompassed modeling the dopamine D3 receptor co-crystallized with the antagonist eticlopride [9] (Figure 1). The dopamine D3 receptor is closely related to the adrenergic beta 1 and 2 receptors, for which a crystal structure has already been elucidated [11,12]. The aminergic receptor family possesses a high sequence identity for the residues involved in ligand binding, including D3.32, S5.43, S5.46, and Y7.43 [13-16]. Due to the functional similarity and identical binding sites of the target to the adrenergic receptors, it was considered as the easiest of the three challenges.

The second case encompassed constructing a model for the chemokine receptor CXCR4 co-crystallized with a small ligand 1T [17] (Figure 1). The similarity of the chemokine receptor CXCR4 in sequence and function is distant compared to the GPCRs for which a crystal structure has been elucidated [10] and as such was expected to pose a more difficult challenge than the dopamine D3 receptor complex. The importance of certain amino acids for the binding of small ligands has been described [18-20], and the ligand in question is one from a compound series for which structure activity relationship data has been determined [17]. These data could be used to derive binding hypotheses for the ligand in the CXCR4 model.

The third case involved building a model for the chemokine receptor CXCR4 co-crystallized with a large cyclic peptide, the antagonist CVX15 [21,22] (Figure 1). Due to the large conformational space accessible to the ligand, it was considered the most difficult challenge in GPCR Dock 2010: none of the deposited models were able to predict any of the critical contacts between protein and ligand [6]. As the current review focuses on the prediction of interactions between proteins and small (drug-like) ligands, the CVX15 modeling case will not be described in this review.

### 2.2. Modeling Approaches

**Figure 2:**
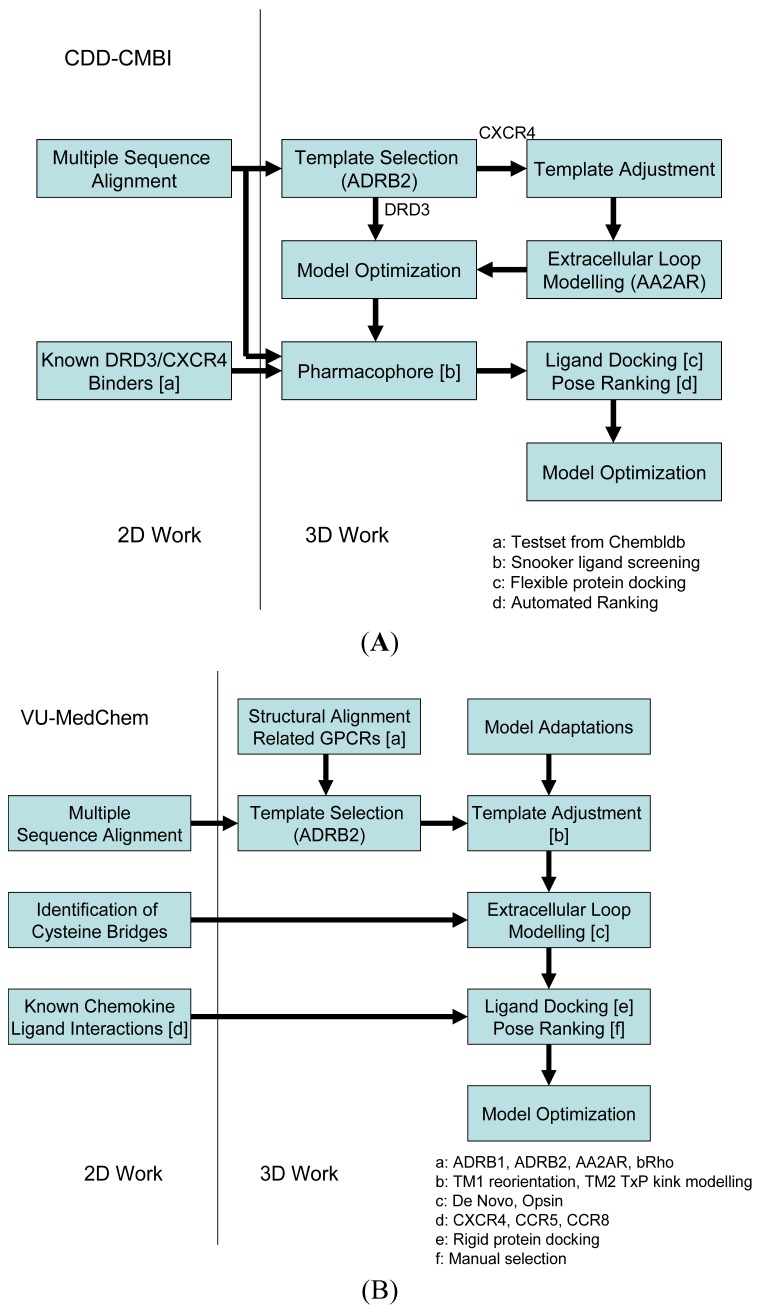
Modeling workflows as used by (**A**) the CDD-CMBI group; and (**B**) the VU-MedChem group in the GPCR Dock 2010 challenge.

Our two research groups have taken different approaches to construct the structural models for the challenge. The CDD-CMBI group contested in both small ligand challenges and ranked second for the dopamine D3 receptor and fourteenth for the chemokine CXCR4 receptor, whereas the VU-MedChem group only contested in the CXCR4-1t challenge for which they obtained first rank [6]. The CDD-CMBI group chose to combine sequence conservation knowledge with the placement of a pharmacophore definition as a description for the protein-ligand interactions in space (Figure 2A). In parallel, a homology model was constructed based on a template structure, which was subsequently combined with the pharmacophore and flexible receptor docking to obtain a structural model for the protein-ligand interactions. The VU-MedChem group constructed a structural model after which literature data was used to determine the most likely protein-ligand interactions (Figure 2B). Each of the steps taken by our groups is graphically outlined below.

### 2.3. Sequence Analysis

#### 2.3.1. Current Approaches

The construction of a multiple sequence alignment of the target sequence with a series of potential homologues is an important step in predicting the structure of the target protein [2]. Potential misalignments have a direct impact on the location of amino acids in the protein model, which in turn directly influences the models ability to correctly predict protein-ligand interactions. As such, the accuracy of the alignment should be optimized as much as possible. To accomplish this, common practice is to include sequences belonging to target family members in an attempt to identify important residues and sequence motifs that might point to a similarity in protein fold or function. Highly conserved amino acids indicate the conservation of the general protein function, three-dimensional fold and structural features. Amino acids that are very different between homologous proteins potentially indicate locations of specificity to binding partners such as small ligands or other proteins.

A G protein-coupled receptor contains seven transmembrane helices, each of which contains highly conserved residues and sequence motifs (DRY in TM3, CWxP in TM6 and NPxxY in TM7) likely related to generic receptor activation. Based on sequence and motif conservation, Ballesteros and Weinstein [23] developed a generic numbering scheme for GPCRs. This numbering scheme allows for consistent residue numbering across multiple proteins, independent of their sequential numbers. The underlying principle is that residues with the same general residue number have equivalent locations in their tertiary structures and consequently in the multiple sequence alignments. Residue numbers are in the format ‘X.Y’, where X indicates the TM helix, and Y the residue position with respect to the most conserved residue position in the helix, which gets the number 50. In addition to these conserved residues, most class A GPCRs also contain conserved cysteine residues in both TM3 (C3.25) and the extracellular loop 2 (C45.50) that together form a cysteine bridge. Resulting from these observations, the alignment of the structural aspects for GPCRs is most challenging for residues arranged outside of the transmembrane helices, in the N-terminus, intracellular and extracellular loops, and the C-terminus. However, most GPCR ligands are interact with the transmembrane domain [24], making the construction of a protein-ligand binding model feasible.

#### 2.3.2. DRD3 Case

Of the crystallized GPCRs the adrenergic beta 2 receptor (ADRB2) has the highest sequence similarity with the dopamine D3 receptor, making this the most suitable template for modeling studies. The alignment of the dopamine D3 sequence with the ADRB2 sequence was based on alignments from the GPCRDB [25], with additional manual refinements in the loop regions. A sequence-based prediction of residues involved in ligand interaction was made for all transmembrane residues. Residues were scored according to ligand binding probability based on an analysis of Shannon entropies of residue positions [26] of a multiple sequence alignment of around 7700 class A GPCR transmembrane domains. The alignment included 64 dopamine receptor D2 and D3 sequences of in total 34 species. The most important ligand interacting residues were predicted to be D3.32, V3.33, S5.42, H6.55, Y7.35 and T7.39, which is corroborated by mutation data [14-16].

#### 2.3.3. CXCR4 Case

**Figure 3:**
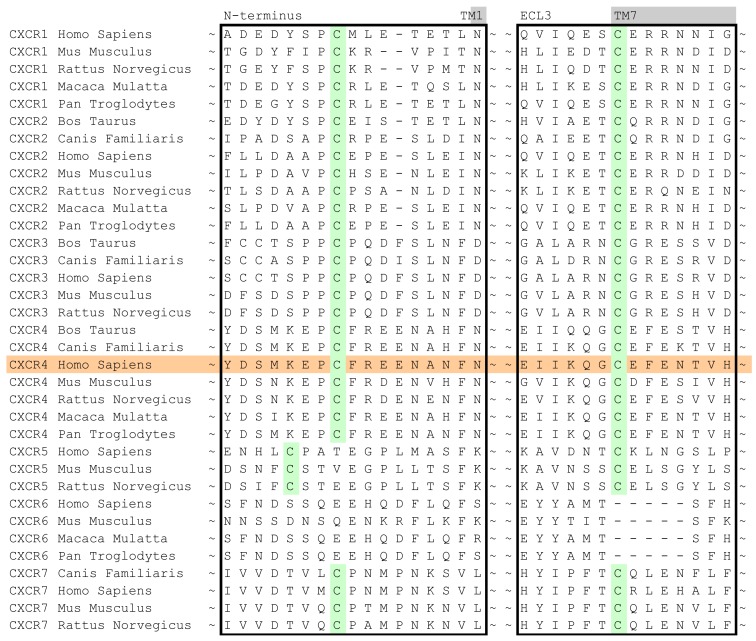
Sequence alignment of the CXCR family including various species. Indicated are the conserved cysteine residues in the N-terminus and the extracellular loop 3 which are hypothesized to form a cysteine bridge.

In case of the CXCR4 challenge, the multiple sequence alignment study was supplemented with a literature study in order to investigate the target for conservation, specific family motifs and the importance of amino acids involved with ligand binding. A sequence alignment of all CXC chemokine receptors was constructed by sequence retrieval from the Uniprot database [27] and alignment with ClustalW [28]. The likelihood of cysteine bridges was assessed based on their sequence conservation (Figure 3). Similar to all other class A GPCRs, CXCR4 contains the cysteine residues C3.25 and C45.50. However, the sequence analysis also showed that all CXCR isoforms except for CXCR6 contain a cysteine residue both in the N-terminus as well as the extracellular loop 3. From these observations, it was concluded that for CXCR4 an additional cysteine bridge should be incorporated into the protein model between the N-terminus and ECL3. The presence of both cysteine bridges were indeed confirmed by the CXCR4 crystal structures [10].

### 2.4. Template Selection and Construction

#### 2.4.1. Current Approaches

Template selection is mostly based on the sequence similarity between the template structure and the target sequence. If the sequence identity is low (< 20%) and the structural aspects of the model are not highly conserved (alpha-helices and beta-sheets), it is very challenging to construct a predictive model for the protein fold [2] and residues interacting with potential ligands. For GPCRs, the crystal structures available for various proteins [8,11,12,29] have shown only slight differences in the general arrangement of the seven transmembrane helices. The only marked differences were observed when comparing the activated [29] and inactivated [30] conformations of the rhodopsin structure. Here, a reorientation of amino acids in the central and intracellular regions of the TM region as well as a twisting of TM5 and TM6 are observed. Currently, novel insights in the conformational changes upon activation can also be derived from the activated AA2AR [31] and ADRB2 [32,33] structures.

Due to the structural conservation, model construction based on any of the crystal structures would likely result in a preliminary model of the transmembrane region with most of the amino acids correctly pointing into the helical bundle [1,24]. Minor adjustments can be made to the alpha helices in case gaps, insertions or helical kinks are expected. This holds particularly true for the occurrence of proline residues in helices combined with either serine or threonine TM residues [34,35]. Adjustments of the template to accommodate these differences can be accomplished by constructing a custom template for the helix and using molecular dynamics or Monte Carlo simulations to predict the overall helical fold.

With regard to the loops, there are noticeable differences, especially in the second extracellular loop, which seems to show a different fold in all currently known protein families. In bovine rhodopsin this loop folds into a beta sheet structure, the adrenergic beta 1 and 2 receptors exhibit an alpha helical fold, and the adenosine A2A receptor shows a coil structure. Using experimental mutation data on the extracellular loop residues, one can produce a protein-ligand interaction model and restrain amino acids from the loop in the binding site while optimizing the rest of the loop structure [36-39]. However, when no data is available, it is advised to omit the modeling of the extracellular loops altogether and prioritize the modeling of interactions with residues in the TM bundle. Multiple template structures can be used as a basis for the construction of the final model, such as a combination of a template for the transmembrane domain and a template for the loop regions.

#### 2.4.2. DRD3 Case

Since it has the highest similarity to the dopamine D3 receptor, the ADRB2 structure [11] (PDB code 2RH1) was chosen as the modeling template. Since the residues in the loop between TM5 and TM6 (residues 218-317) were not present in the crystal structure due to the insertion of T4 lysozyme, these residues were discarded in the modeling process. No additional modifications to the structure were deemed necessary based on the sequence.

#### 2.4.3. CXCR4 Case

**Figure 4:**
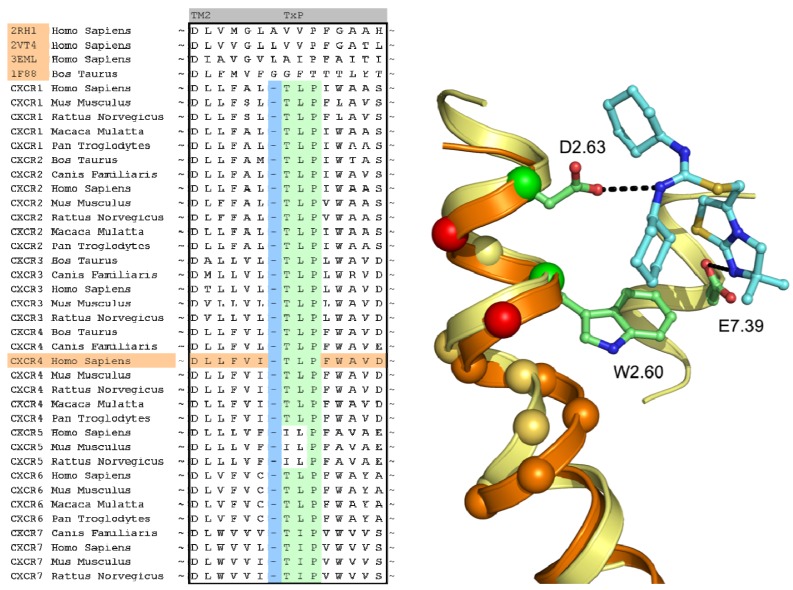
Sequence conservation of the TxP motif in the CXCR family and the implication of a possible misalignment on the amino acids pointing into the TM bundle. Using the unadjusted TM2 template of ADRB2 (orange ribbon and C_alpha_ spheres) would place W2.60 and D2.63 on the red C_alpha_ spheres, where the residues would not contact the ligand. In the CXCR4 crystal structure (yellow ribbon and C_alpha_ spheres), W2.60 and D2.63 are positioned on the green C_alpha_ spheres, indicating a shift from the ADRB2 template.

A structural assessment of available crystal structures of other G protein-coupled receptors was ensued following the alignment of their sequences to those of the chemokine receptor family. The structural alignment was performed with MOE2009.10 [40] and consisted of the structures of the human adenosine A2A Receptor [8] (PDB: 3EML), human adrenergic Beta 1 Receptor [12] (PDB: 2VT4), human Adrenergic Beta 2 receptor [11] (PDB: 2RH1) and the bovine opsin structure [29] (PDB: 3DQB). The selection and construction of a template structure posed several challenges. Overall, none of the sequences of the crystal structures possessed a significantly higher sequence similarity with the CXCR4 sequence than another, and as such, the choice of a template structure seemed arbitrary. In the end, the crystal structure of ADRB2 was found to be a feasible template due to the importance of D3.32, N7.39 and Y7.43 in the binding of the antagonist carazolol [11], and the analogous involvement of residues at these positions in chemokine receptor binding by small ligands [20,41-43]. From the sequence analysis and literature [44] it became clear that the chemokine receptor family possessed a unique TxP motif in TM2 which when aligned to the available crystal structures would produce a gap at the top of the transmembrane helix (Figure 4). A misalignment of this region would not put important amino acids into the TM bundle such as D2.63 [20]. To overcome this problem, we decided to customize the conformation of TM2 to accommodate the difference in amino acid rather than using an existing crystal structure as a template. In a manner similar to Govaerts *et al.* [44], a three-dimensional model of the helix was constructed to predict the helical bend of the TxP motif using the AMBER program [45] which was subsequently incorporated into the template. Lastly, TM1 of the template structure was oriented closer to TM7 since there is a spatial gap between these helices in the crystal structure due to the uncapped N-terminus of TM1. It was reasoned that the smaller residue at position 7.40 in CXCR4 compared to that of in the ADRB2 structure (A *vs.* W, respectively) could accommodate the helical repositioning.

### 2.5. Homology Model Construction

#### 2.5.1. Current Approaches

There are many homology modeling software packages available that allow the user to construct a three-dimensional model for their desired target sequence based on a template structure. Most packages work in a similar fashion. Residues that are in common between the template and the target are typically kept unaltered in the initial step of the model construction. Next, residues that differ are mutated and placed in an initial conformation based on a rotamer library [46,47] that consists of the most commonly observed orientations of the residues throughout various crystal structures in the Protein Data Bank [48]. Residues are perturbed and reoriented whilst decreasing the amount of steric clashes and increasing the amount of stabilizing interactions. Finally, multiple different homology models are created and if desired optimized by the modeling program. It is recommended to investigate the predictive value of the model by identifying potential protein-ligand interactions as hypothesized by the user or as determined from literature data, as described below.

#### 2.5.2. DRD3 Case

The template was prepared by a cleanup of the structure (removal of waters, sulfate ions, maltose, acetamide and butanediol), and removal of the T4 lysozyme protein. The lipids were retained for modeling. Using the alignment and the template as the starting point, modeling was performed with the Yasara program and its built-in modeling algorithm [49]. Side chains were added with Yasaras implementation of SCWRL [50], and then the model was subjected to an energy minimization with the Yasara2 force field as described previously [49]. WHAT CHECK [51] validation scores were used to score and rank the final models. The generated model was refined manually and a final energy minimization step was applied to relax the atoms of the DRD3 model.

#### 2.5.3. CXCR4 Case

Structural modeling of the CXCR4 structure was commenced with the TM bundle and most of the loop structures. Three structural regions were excluded from the initial model and were constructed later. These included the extracellular loop 2 (ECL2), the N-terminus, and the C-terminus. The extracellular loop 2 could not be modeled using any of the protein crystal structures available since the length of the CXCR4 loop is different and amino acids would either have to be added to or deleted from the (template) sequence. Different conformations of ECL2 were generated using MODELLER [52] and incorporated into the CXCR4 models, and one conformation was constructed based on the ECL2 of the opsin structure [29] (PDB: 3DQB). A structural model for the N-terminus could be retrieved from the crystal structure of the ligand CXCL12 [53] (PDB: 2K04) after positioning the arrangement on top of the TM bundle of the CXCR4 model. The C-terminus was modeled with a random arrangement since all atoms were requested for submission in the GPCR Dock assessment.

### 2.6. Ligand Interaction Modeling

#### 2.6.1. Current Approaches

The prediction of protein-ligand interactions can be a challenging task for which the availability of experimental data is often beneficial. Many different experimental methods have been applied to identify GPCR ligand binding sites, and elucidate protein conformations and protein-ligand interactions, including site-directed mutagenesis studies [54], infrared probes [55], NMR spectroscopy [56], fluorescence measurements [57,58], and the use of unnatural amino acids [59] and amino acid chelators [43]. Each of these tools provides information about amino acids interacting either with each other or with the ligand. In addition to these pharmacological and biophysical methods, a common method is the evaluation of other receptor binders and structural analogues of the ligand with regard to their interaction with the protein [17,60] (and protein mutants if available). The majority of the methods allow the identification of residues interacting with the ligand thereby providing an anchor for the arrangement of the ligand in the binding site. Thus model validation can be performed by investigating both structure activity relationships based on ligand analogues, but it can also utilize site-directed mutagenesis data.

Molecular docking methods can be used to generate an initial binding pose of the small molecule ligand in the protein model. Docking programs rank the poses based on a scoring function that is knowledge-based, energy-based or empirical [5]. Since the scoring functions are optimized for protein-ligand complexes such as in the Protein Data Bank, one may assume that the results are precise. However, when little is known about the protein binding cavity or the rotamer conformations of the amino acids in the binding pocket, one cannot be certain that the docking program will generate or select the correct binding mode. In such case, it is helpful to post-process the binding poses using filtering schemes that prioritize specific residue interactions [1].

#### 2.6.2. DRD3 Case

**Figure 5:**
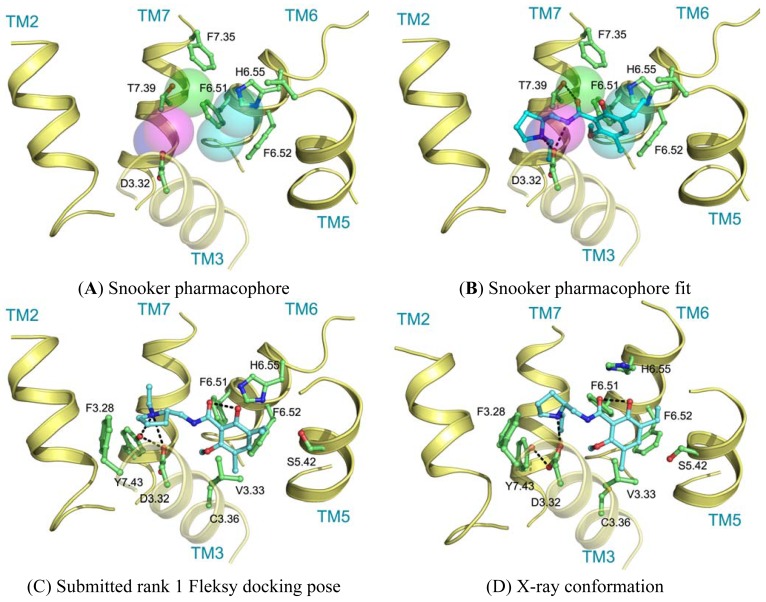
Binding pose as predicted by CDD-CMBI using Snooker and Fleksy compared to the X-ray structure.

Ligand interactions were modeled using pharmacophore searches followed by flexible receptor docking. Structure-based pharmacophores derived from the transmembrane domains of the constructed homology models were built using the Snooker program [61]. In short, the Snooker method scores residue positions based on sequence conservation in a multiple sequence alignment, and deduces the key interacting residues from the derived statistics. Known DRD3 actives were retrieved from Chembl [62] using a 50 nM activity cutoff. Conformations of the ligands were generated using Cyndi [63]. A pharmacophore search was performed to identify the structure-based pharmacophore complementary to most of the active compounds. The donor and positive ionizable features in the resulting pharmacophore originate from D3.32, the acceptor feature from T7.39, and the hydrophobic features from F6.51, F6.52, H6.55 and F7.35. Subsequently, eticlopride was matched in this structure-based pharmacophore (Figure 5A, B). The low-resolution binding modes obtained from Snooker were used to guide high-resolution molecular docking by the Fleksy program [64]. The various orientations of eticlopride in the DRD3 receptor model were used as anchors to guide induced fit docking using the Fleksy protocol. The generated poses were optimized in the homology model (Figure 5C) and ranked using a consensus scoring function which utilizes docking scores, geometrical quality indicators and molecular dynamics force field interaction energies. All final poses contained the charged interaction of the basic amine with D3.32 and the hydrophobic interactions with TM3, TM5, TM6 and TM7 similarly to carazolol in the ADRB2 receptor complex and timolol in the ADRB1 receptor complex. A retrospective comparison to the DRD3 crystal structure revealed that the polar interaction with D3.32 was correctly predicted, as well as the hydrophobic contacts to V3.33, V5.39, V5.39, H6.55, F7.35, T7.39 and Y7.43 (Figure 5C, D). Many of these residues are in agreement with the pharmacophore features defined by the Snooker protocol.

**Figure 6:**
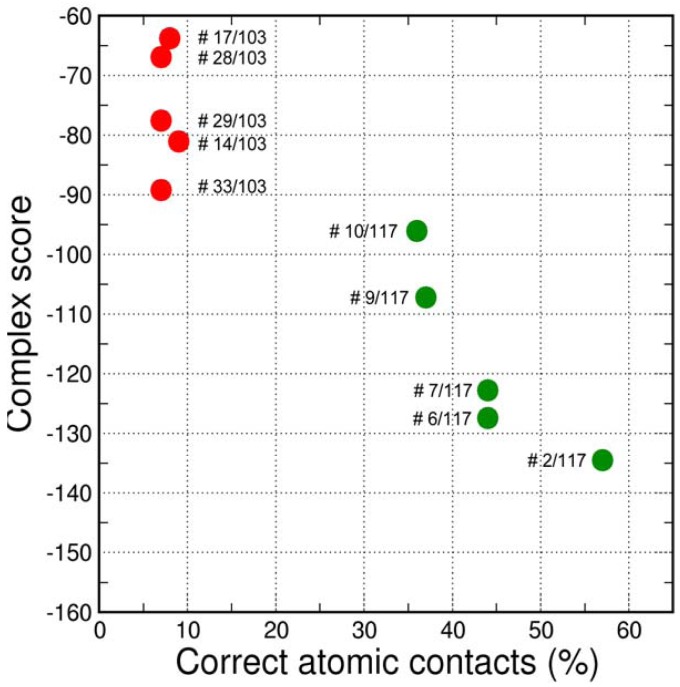
Ranking of the submitted models by the Fleksy consensus score. The five submitted models for DRD3 are shown in green, for comparison the five submitted models by CDD-CMBI for CXCR4 are shown in red. The consensus complex score (lower is better) is plotted against the percentage of correct contacts as determined in the final GPCR dock 2010 assessment. The final rank in the GPCR dock 2010 assessment is indicated for each of the solutions.

The final GPCR Dock 2010 assessment of the five submitted models placed each of them in the top 10 of all 117 submitted models with the best model ranking second. Despite a relatively high receptor model RMSD the submitted models were able to capture a large part (35% to 57%) of the receptor-ligand atomic contacts, including the hydrogen bond with D3.32 and the hydrophobic interactions with F6.51, F6.52, H6.55 and F7.35. The best binding mode was able to capture 36 of 65 atomic contacts as well as 12 of 15 residues directly interacting with the ligand. Interestingly, the accuracy of the five submitted DR3D models is in excellent agreement with the ranking generated by the Fleksy consensus scoring function [6] (Figure 6). This highlights the potential of knowledge based scoring functions in the identification of near-native receptor-ligand complex geometries. The scoring function performs less well in ranking the much less accurate solutions generated by CDD-CMBI for the CXCR4 target (Figure 6), but does correctly assign them a worse score compared to the DRD3 solutions.

#### 2.6.3. CXCR4 Case

**Figure 7:**
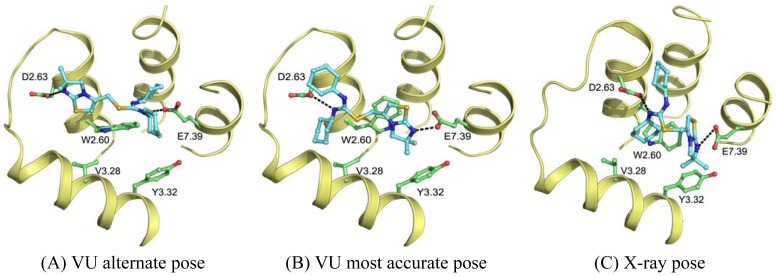
Binding poses as predicted by VU-MedChem using GOLD compared to the X-ray structure.

Wong [20] and Rosenkilde [43] determined that negatively the charged residues D2.63, D4.60, D6.58 and E7.39 play a role in the CXCR4 binding of several antagonists. Based on the physico-chemical properties of ligand 1T (Figure 1), it seemed likely protonated on one of the thiourea moieties. As such, the negatively charged residues were the first candidates for an anchor point of the ligand in the TM bundle of CXCR4. Eight possible binding poses were conceived in which the ligand is sandwiched between two negatively charged residues that interact with either of the thiourea moieties (D2.63 was only able to combine with E7.39). From these poses, the five poses that corroborated the structure-activity relationships as described by Thoma *et al.* [17], were chosen for the final models. The ligands were docked into the models using GOLD [65] using the restraints of ionic interactions. Both binding modes interacting with D2.63 and E7.39 were among those chosen for submission to GPCR Dock 2010 (Figure 7A, B). The final models were optimized using AMBER molecular dynamics simulation including the ionic interactions, followed by an unconstrained energy minimization and molecular dynamics to optimize the overall structure. Model ranking was performed by visual inspection.

Compared to the crystal structure, the 5th ranked model possessed the most similar binding mode in the CXCR4 structure. In total, 19 of 64 atomic contacts from five of 13 residues were correctly predicted by the model. Although this might seem a low amount, the model did capture the hydrophilic interactions of the ligand with D2.63 and E7.39 (Figure 7B, C), and the fraction of the pocket that was predicted correctly reached 45% [6]. The major discrepancy between model and crystal structure is the folding of ECL1 and ECL2 to place the correct residues in the binding cavity.

The other research groups that participated in the GPCR Dock 2010 were able to reproduce the interaction of 1t with either D2.63 or E7.39, but were unable to produce a pose that interacted with both ionic residues. The explanation for this observation can be twofold. Firstly, the location of the ligand 1t in the transmembrane domain is new, namely, inside the minor pocket [24], whereas all potential template structures portrayed their ligand in the major pocket. Secondly, the correct spatial construction of the binding cavity is dependent on the sequence alignment and resolving the influence of the TxP motif on TM2, as well as the positioning of TM1 due to the presence of the cysteine bridge between the N-terminus and ECL3 [10]. The VU-MedChem group was able to capture the correct interactions in the binding cavity because they focused their attention on these particular regions.

#### 2.6.4. Water Molecules

Although none of the modeling attempts have included the prediction of protein-water or ligand-water interactions, it should be noted that conserved water clusters have been identified for class A GPCRs between TM1, TM2, TM6 and TM7. These conserved waters are suggested to be involved in receptor activation [66] and can in principle be included in GPCR modeling procedures [67]. However, none of these water molecules is directly involved in water-mediated protein-ligand interactions. In the AA2AR crystal structures, water molecules are included in protein-ligand hydrogen bonding networks [8] and consideration of some of these water molecules has been shown to improve structure-based virtual screening accuracy [68]. In the ADRB1, ADRB2 and DRD3 crystal structures, no water molecules have been resolved in the vicinity of the ligand, and none of the crystallographic water molecules in contact distance of the ligand in the CXCR4 structure mediate polar protein-ligand interactions. Moreover, given the hydrophobicity of the CXCR4 pocket, it is unclear to which extent the water molecules influence ligand binding; hence it would have been difficult to predict the position of the water molecules in the pocket. Finally, the need to include water molecules in the prediction of protein-ligand interactions is target-dependent, as demonstrated by comparative docking studies [69,70].

## 3. Conclusions

Structure-based modeling and design can aid in understanding and optimizing protein-ligand interactions and as such has proven a valuable tool in modern drug discovery. The approaches to structure-based modeling have evolved to include prior knowledge, which greatly aids the identification of ligand binding cavities as well as the validation of generated ligand binding poses. In the GPCR Dock 2010 challenge, our groups used different approaches to obtain predictions for the protein-ligand interactions of eticlopride and 1t in the DRD3 and CXCR4 crystal structure, respectively.

The largely automated use of extensive sequence analysis in the derivation of the structure-based pharmacophores followed by the selection of the structure-based pharmacophore best correlating to a large number of known actives, resulted in a pharmacophore definition which correctly encoded the crucial protein ligand interactions for DRD3. The simultaneous optimization of protein-ligand interactions and the protein and ligand structure themselves in a restraint docking procedure allowed that correct atomic contacts were produced and that these predicted contacts were optimized. Importantly, the applied knowledge based scoring function was able to correctly identify and assign the highest rank to the best near-native complex geometry without manual intervention.

Resulting from a knowledge-based investigation of the CXCR sequence, the CXCR4 model required a template adjustment in TM1 and TM2 to accommodate an expected cysteine bridge in the N-terminus as well as a kink in TM2. Inclusion of such detail resulted in a highly customized CXCR4 model which was able to correctly capture the most important protein-ligand interactions as observed in the crystal structure. Without in depth sequence analysis, experimental receptor knowledge and knowledge on ligand analogues to validate and refine the ligand binding prediction, the result would not have been as successful.

From this study we conclude that the integration of experimental target data and in silico studies in iterative cycles is of prime importance for the accurate prediction of the protein architecture as well as ligand binding modes therein. Even then, the elucidation of a ligand binding mode is not always clear cut, since symmetry in the ligand (such as for 1t) or binding pocket increases modeling difficulty. In the future, the inclusion of experimental data and further development of more automated procedures will help to fill the gaps that still exist in the GPCR structural landscape, even when GPCRs belonging to different subfamilies are being resolved by crystallography in rapid succession (http://gpcr.scripps.edu/) [31-33,71].
